# Effect of tofacitinib on dactylitis and patient-reported outcomes in patients with active psoriatic arthritis: post-hoc analysis of phase III studies

**DOI:** 10.1186/s41927-022-00298-4

**Published:** 2022-09-01

**Authors:** Ana-Maria Orbai, Philip J. Mease, Philip S. Helliwell, Oliver FitzGerald, Dona L. Fleishaker, Rajiv Mundayat, Pamela Young

**Affiliations:** 1 Division of Rheumatology, Johns Hopkins University School of Medicine, Baltimore, MD USA; 2 Rheumatology Research, Swedish Medical Center, and University of Washington School of Medicine, Seattle, WA USA; 3 Leeds Institute of Rheumatic and Musculoskeletal Medicine, University of Leeds, Leeds, UK; 4 Conway Institute for Biomolecular Research, University College Dublin, Dublin, Ireland; 5 Pfizer Inc, Groton, CT USA; 6 Pfizer Inc, New York, NY USA; 7 Pfizer Inc, Collegeville, PA USA

**Keywords:** Spondyloarthritis, Psoriatic arthritis, Patient-reported outcomes, Dactylitis, Tofacitinib

## Abstract

**Background:**

Tofacitinib is an oral Janus kinase inhibitor for the treatment of psoriatic arthritis (PsA). This post-hoc analysis of two phase III studies in patients with PsA treated with tofacitinib assessed dactylitis by location, and the impact on patient-reported outcomes (PROs).

**Methods:**

Patients received tofacitinib 5 or 10 mg twice daily (BID), or placebo. Endpoints included change from baseline in Dactylitis Severity Score (DSS), proportions of patients with dactylitis, Psoriatic Arthritis Disease Activity Score (PASDAS), and PROs (Health Assessment Questionnaire-Disability Index [HAQ-DI]; Functional Assessment of Chronic Illness Therapy-Fatigue [FACIT-F]; Short Form-36 Health Survey [SF-36] Physical Component Summary [PCS], Mental Component Summary [MCS], and physical functioning [PF]; arthritis pain; and Work Limitations Questionnaire [WLQ]). Descriptive statistics were generated by visit and treatment. Change from baseline in PROs were evaluated by multivariate linear regression.

**Results:**

The analysis included 373/337 patients with baseline DSS > 0/DSS = 0. Regardless of location, DSS improvements in patients with DSS > 0 were greater from month 1 with tofacitinib (10 mg BID) versus placebo. For patients with DSS > 0/DSS = 0, both doses of tofacitinib led to mean dactylitis presence ≤ 15%/< 2% for all digits at month 6, and PASDAS (by dactylitis location) was lower versus placebo at month 3. Dactylitis location was not significantly associated with change from baseline in PROs.

**Conclusion:**

Tofacitinib resulted in sustained improvements in dactylitis irrespective of location, with minimal emergence of new dactylitis.

*Trial registration* NCT01877668; NCT01882439.

**Supplementary Information:**

The online version contains supplementary material available at 10.1186/s41927-022-00298-4.

## Background

Psoriatic arthritis (PsA) is a chronic, immune-mediated disease that has multiple manifestations, including inflammation of the peripheral joints, tendons, ligaments, skin, and the axial skeleton [1–3]. It is associated with pain, increased levels of fatigue, impaired physical function, and reduced work productivity, which can have a substantial impact on patients’ health-related quality of life (HRQoL) [1–5].

Dactylitis is a diffuse and, in its acute phase, painful swelling of the fingers and/or toes, and is considered a hallmark of PsA [6, 7]. Up to 50% of patients with PsA may experience dactylitis [6, 8], and it is more common in feet than in hands [8]. Dactylitic digits exhibit a greater degree of radiological damage than digits unaffected by dactylitis [6, 8]. As such, dactylitis is considered a core domain of musculoskeletal symptoms in PsA [9] and is important for both patients and physicians when developing treatment strategies. Disease-modifying antirheumatic drugs (DMARDs) are recommended as first-line treatments for dactylitis [10]. There is evidence supporting the efficacy of biologic DMARDs (bDMARDs), including tumor necrosis factor inhibitors (TNFi), and inhibitors of interleukin (IL)-17 and IL-23, for the treatment of dactylitis [7, 10–14]. However, effective therapies to treat dactylitis in patients with PsA are still needed.

Tofacitinib is an oral Janus kinase inhibitor for the treatment of PsA. The efficacy and safety of tofacitinib 5 mg twice daily (BID; recommended dosage) [15, 16] and 10 mg BID has been demonstrated in phase III randomized controlled trials (RCTs) of patients with active PsA with an inadequate response to either conventional synthetic DMARDs (csDMARDs) (OPAL Broaden; NCT01877668) [17] or TNFi therapy (OPAL Beyond; NCT01882439) [18], and were investigated in an open-label, long-term extension study (OPAL Balance; NCT01976364) [19].

In phase III RCTs of patients with active PsA, tofacitinib treatment was associated with greater improvements in Dactylitis Severity Score (DSS) than placebo, at month 3 [17, 18]. This post-hoc analysis of data pooled from phase III RCTs further explored the effects of tofacitinib on dactylitis, including the effect on individual digits, and the impact of tofacitinib treatment on patient-reported outcomes (PROs) in patients with PsA with dactylitis, stratified by dactylitis location, compared with patients without dactylitis.

## Methods

### Study design

This post-hoc analysis included pooled data from two phase III studies. OPAL Broaden (NCT01877668) was a 12-month, phase III RCT of tofacitinib in patients with active PsA with an inadequate response to csDMARDs. Patients received tofacitinib 5 or 10 mg BID, placebo (to month 3 only; patients receiving placebo advanced to either tofacitinib 5 or 10 mg BID at month 3), or adalimumab 40 mg subcutaneous injection once every 2 weeks [17]. OPAL Beyond (NCT01882439) was a 6-month, phase III RCT of tofacitinib in patients with active PsA with an inadequate response to TNFi. Patients received tofacitinib 5 or 10 mg BID, or placebo (to month 3 only) [18].

This analysis included patients with PsA receiving tofacitinib 5 mg BID (approved dose) or tofacitinib 10 mg BID from baseline to month 6, or placebo to month 3 [17, 18]. Patients were also treated with a single csDMARD throughout.

Both studies were conducted in accordance with the International Council for Harmonisation Good Clinical Practice Guidelines and the principles of the Declaration of Helsinki. The study protocols were approved by the Institutional Review Board or Independent Ethics Committee at each center, and all patients provided written informed consent.

### Assessments

Patients were categorized at baseline by the presence or absence of dactylitis in the hands and/or feet. Dactylitis was defined as the painful swelling of an entire digit, based on the investigator’s judgment. The number of digits in the hands and feet with dactylitis was evaluated by a blinded, qualified assessor. For each digit of the hands and feet, the severity of dactylitis was scored on a scale of 0–3 (0 = no tenderness; 3 = extreme tenderness). DSS was calculated as the sum of the individual scores for each digit of the hands and feet, which ranged from 0 to 60 (60 = highest dactylitis severity) [20].

Endpoints in this analysis included the number of dactylitic digits per patient, and the proportion of patients with dactylitis in individual digits at months 1 (first post-baseline assessment), 3, and 6, and change from baseline in DSS in patients with DSS > 0 at baseline, and the development of dactylitis through month 6 in patients with DSS = 0 at baseline. Disease activity at baseline and at months 1, 3, and 6 was assessed by Psoriatic Arthritis Disease Activity Score (PASDAS), a composite measure of disease activity, that includes assessments of tender/swollen joints, dactylitis, and enthesitis; Short Form-36 Health Survey (SF-36) Physical Component Summary (PCS); acute-phase response; and patient and physician global assessments (range 0–10; higher scores indicate higher disease activity) [21, 22].

PROs assessed at months 1, 3, and 6 included change from baseline in: Health Assessment Questionnaire-Disability Index scores (HAQ-DI; range 0–3, higher scores indicate greater disability) [23]; HAQ-DI response rate (defined as a ≥ 0.35-point decrease from baseline in HAQ-DI score) [24]; Functional Assessment of Chronic Illness Therapy-Fatigue (FACIT-F) total score (range 0–52; higher scores indicate less fatigue) [25]; SF-36 PCS, Mental Component Summary (MCS), and physical functioning (PF) sub-scores (norm-based scale; higher scores indicate better HRQoL) [26–28]; arthritis pain (assessed by Visual Analog Scale [VAS], range 0–100 mm) [29]; and Work Limitations Questionnaire (WLQ) time management, physical demands, mental/interpersonal demands, and output demands scores (assessed at months 3 and 6 only, range 0–100; higher scores indicate greater work limitation/productivity loss) [30, 31].

### Statistical analyses

Patient demographic and baseline disease characteristics were reported for all patients who received ≥ 1 dose of study treatment. Descriptive statistics were generated for PROs by visit and for each treatment arm, stratified by the presence of dactylitis. Binary endpoints were analyzed using Cochran–Mantel–Haenszel statistics, with non-responder imputation for missing values; 95% confidence intervals (CIs) were derived based on the normal approximation. When comparing with placebo, tofacitinib responses were defined as “greater”, “lower”, or “higher” if the corresponding 95% CIs did not overlap.

The effects of baseline dactylitis on change from baseline in HAQ-DI, SF-36 PF sub-score, and WLQ domain scores at months 3 and 6 were evaluated by multivariate linear regression analysis in patients with DSS > 0 receiving tofacitinib, using backward selection criteria. The baseline parameters included in the model were the respective PRO being assessed (HAQ-DI, SF-36 PF score, or WLQ dimension sub-scores); dactylitis in the right hand (yes/no), left hand (yes/no), right foot (yes/no), and left foot (yes/no); a term for tofacitinib dose was also included in the model. In this analysis, a *P* value of < 0.05 was considered statistically significant. No adjustments were made for multiple comparisons.

## Results

### Patient demographics and baseline disease characteristics

Overall, 710 patients were included in this analysis, of whom 337 (47.5%) had DSS = 0 at baseline, and 373 (52.5%) had DSS > 0 at baseline. Of those patients with DSS > 0 at baseline, dactylitis in the hands or feet was assessed; 251 (67.3%) patients had dactylitis in the hands only, 275 (73.7%) patients had dactylitis in the feet only, and 153 (41.0%) patients had dactylitis in both the hands and feet.

Patient demographics and baseline disease characteristics for patients with DSS > 0 and DSS = 0 are shown in Table 1. At baseline, patient demographics and disease characteristics were generally similar across dactylitis groups and treatment groups; however, of those patients with DSS > 0, a higher proportion of patients with dactylitis versus no dactylitis at baseline were men (e.g., in the hands and feet combined group: DSS > 0, 45.3–65.2%; DSS = 0, 36.0–42.3%). Baseline PASDAS across treatment groups was generally higher in patients with DSS > 0 (6.5–7.1), compared with patients with DSS = 0 (5.4–5.7). Baseline PROs were similar across dactylitis groups and treatment groups.

**Table 1:** Demographics and baseline characteristics in patients with baseline DSS > 0 or DSS = 0

	DSS > 0				DSS = 0 (N1 = 337)
	Hands only (N1 = 251)	Feet only (N1 = 275)	Hands and feet (N1 = 153)	Hands or feet (N1 = 373)	
Tofacitinib 5 mg BID	N = 84^a^	N = 97	N = 54	N = 127^a^	N = 111^b^
Tofacitinib 10 mg BID	N = 87	N = 91	N = 53	N = 125	N = 111
*Placebo*	*N* = *80*	*N* = *87*	*N* = *46*	*N* = *121*	*N* = *115*
Men, n (%)	44 (52.4)	57 (58.8)	31 (57.4)	70 (55.1)	47 (42.3)
	42 (48.3)	42 (46.2)	24 (45.3)	60 (48.0)	40 (36.0)
	*42 (52.5)*	*46 (52.9)*	*30 (65.2)*	*58 (47.9)*	*42 (36.5)*
Age, mean (SD), years	50.3 (12.0)	47.6 (12.5)	48.8 (12.4)	48.9 (12.3)	50.2 (12.5)
	48.8 (11.5)	47.9 (12.0)	48.1 (11.3)	48.5 (12.0)	50.4 (11.4)
	*48.1 (13.8)*	*48.2 (12.7)*	*47.0 (14.5)*	*48.6 (12.7)*	*48.3 (12.3)*
Race, n (%)					
White	80 (95.2)	90 (92.8)	51 (94.4)	119 (93.7)	107 (96.4)
	80 (92.0)	80 (87.9)	49 (92.5)	111 (88.8)	110 (99.1)
	*77 (96.3)*	*83 (95.4)*	*45 (97.8)*	*115 (95.0)*	*107 (93.0)*
Other	4 (4.8)	7 (7.2)	3 (5.6)	8 (6.3)	4 (3.6)
	7 (8.0)	11 (12.1)	4 (7.5)	14 (11.2)	1 (0.9)
	*3 (3.8)*	*4 (4.6)*	*1 (2.2)*	*6 (5.0)*	*8 (7.0)*
BMI, mean (SD), kg/m^2^	29.8 (6.8)	29.7 (6.2)	29.2 (6.4)	30.0 (6.5)	29.6 (6.2)
	29.9 (6.5)	30.1 (6.7)	29.6 (6.9)	30.2 (6.5)	30.3 (6.1)
	*29.5 (5.9)*	*29.5 (5.4)*	*29.9 (5.4)*	*29.3 (5.7)*	*29.1 (5.6)*
PsA duration, mean (SD), years	9.3 (9.3)	7.7 (6.8)	8.2 (8.0)	8.5 (8.2)	8.7 (7.6)
	7.8 (6.2)	8.1 (7.0)	8.1 (6.7)	7.9 (6.5)	7.0 (6.7)
	*7.8 (6.9)*	*8.2 (8.8)*	*7.3 (7.4)*	*8.3 (8.1)*	*7.8 (6.9)*
DSS,^c^ median (range)	7.0 (1.0–52.0)	6.0 (1.0–52.0)	11.0 (3.0–52.0)	6.0 (1.0–52.0)	NA
	10.0 (1.0–40.0)	8.0 (1.0–40.0)	11.0 (2.0–40.0)	6.0 (1.0–40.0)	NA
	*8.0 (1.0–31.0)*	*8.0 (1.0–31.0)*	*12.5 (4.0*–*31.0)*	*6.0 (1.0*–*31.0)*	*NA*
Dactylitic digits count,^c^ median (range)	2.0 (1–10)	2.0 (1–10)	5.0 (2–20)	3.0 (1–20)	NA
	2.0 (1–10)	2.0 (1–10)	5.0 (2–20)	4.0 (1–20)	NA
	*3.0 (1–10)*	*2.0 (1–10)*	*7.0 (2–17)*	*3.0 (1–17)*	*NA*
PASDAS, mean (SD)^d,e^	6.7 (1.1)	6.6 (1.2)	6.9 (1.1)	6.5 (1.2)	5.5 (1.0)
	6.8 (1.1)	6.8 (1.2)	7.1 (1.1)	6.7 (1.1)	5.7 (0.9)
	*6.7 (1.0)*	*6.7 (1.0)*	*7.0 (1.0)*	*6.6 (1.0)*	*5.4 (1.1)*
HAQ-DI, mean (SD)	1.3 (0.6)	1.2 (0.7)	1.3 (0.7)	1.2 (0.7)	1.2 (0.7)^b^
	1.3 (0.6)	1.3 (0.6)	1.3 (0.6)	1.3 (0.6)	1.2 (0.6)
	*1.3 (0.7)*	*1.2 (0.6)*	*1.2 (0.6)*	*1.3 (0.7)*	*1.1 (0.7)*
FACIT-F total score, mean (SD)	25.7 (11.4)	27.5 (11.2)	25.7 (11.4)	27.1 (11.2)	26.8 (11.7)^b^
	26.0 (10.1)	26.3 (10.1)	25.2 (8.8)	26.5 (10.6)	27.5 (10.4)
	*26.5 (11.1)*	*27.6 (10.1)*	*28.1 (10.3)*	*26.7 (10.7)*	*29.3 (10.6)*
SF-36, mean (SD)					
PCS score	34.4 (8.1)^a^	34.0 (8.7)	33.9 (8.7)	34.3 (8.3)^a^	34.5 (8.2)^f^
	33.1 (8.4)	33.1 (7.8)	32.1 (7.1)	33.5 (8.5)	34.1 (8.9)
	*34.4 (8.7)*	*34.3 (8.0)*	*35.1 (8.4)*	*34.0 (8.3)*	*36.6 (8.9)*
MCS, score	38.8 (10.9)^a^	40.9 (12.2)	39.2 (11.1)	40.2 (11.9)^a^	40.2 (11.7)^f^
	39.8 (12.5)	39.3 (11.5)	39.6 (12.2)	39.5 (12.0)	40.7 (12.3)
	*39.0 (10.9)*	*39.7 (11.1)*	*40.1 (10.5)*	*39.1 (11.3)*	*41.2 (12.2)*
PF domain score^g^	33.4 (10.2)	34.1 (10.7)	32.8 (11.1)	34.2 (10.1)	33.6 (10.4)
	34.2 (9.7)	33.8 (9.0)	33.2 (7.7)	34.3 (9.9)	33.8 (10.0)
	*33.4 (11.2)*	*34.2 (9.8)*	*34.3 (10.6)*	*33.6 (10.4)*	*36.7 (9.8)*
Arthritis pain, mean (SD)^h^	59.0 (23.4)	55.2 (23.6)	56.5 (25.1)	57.1 (22.9)	54.8 (24.3)
	58.8 (21.7)	59.2 (21.2)	58.3 (19.8)	59.3 (22.1)	54.9 (22.0)
	*59.8 (23.4)*	*56.5 (22.9)*	*57.8 (23.6)*	*58.2 (23.0)*	*49.8 (25.2)*
WLQ dimension sub-scores, mean (SD)^i^^,^^j^					
Time management	36.4 (24.1)	37.0 (23.8)	38.8 (24.6)	35.9 (23.6)	41.5 (27.9)
	39.1 (24.9)	35.1 (24.7)	37.3 (23.5)	36.7 (25.4)	43.3 (26.8)
	*49.0 (28.2)*	*46.3 (26.8)*	*48.4 (28.6)*	*47.2 (27.0)*	*35.3 (24.0)*
Physical demands	43.4 (23.2)	49.0 (25.5)	46.0 (23.9)	46.7 (25.0)	53.8 (26.7)
	51.9 (24.2)	49.3 (23.6)	49.9 (22.1)	50.7 (24.7)	48.1 (27.0)
	*46.2 (27.9)*	*48.3 (26.1)*	*50.9 (28.4)*	*45.7 (26.2)*	*49.1 (27.0)*
Mental/interpersonal demands	27.6 (22.0)	26.4 (24.2)	28.2 (23.5)	26.4 (23.1)	28.5 (26.9)
	25.5 (20.1)	24.5 (21.3)	24.6 (18.7)	25.1 (21.6)	29.7 (25.3)
	*29.0 (20.5)*	*28.7 (19.9)*	*28.2 (20.9)*	*29.1 (19.9)*	*25.5 (20.2)*
Output demands	30.5 (23.2)	32.2 (24.7)	34.5 (25.3)	30.2 (23.4)	34.2 (27.6)
	33.4 (24.4)	29.9 (23.4)	32.8 (24.2)	30.9 (23.8)	33.3 (28.0)
	*41.1 (28.4)*	*39.5 (25.9)*	*41.3 (27.4)*	*39.8 (27.0)*	*29.2 (20.5)*

*BID* twice daily, *BMI* body mass index, *DSS* Dactylitis Severity Score, *FACIT-F* Functional Assessment of Chronic Illness Therapy-Fatigue, *HAQ-DI* Health Assessment Questionnaire-Disability Index, *MCS* Mental Component Summary, *n* number of patients applicable for each category, *N* total number of patients, *N1* number of patients in a dactylitis group at baseline receiving a particular treatment, *NA* not applicable, *PASDAS* Psoriatic Arthritis Disease Activity Score, *PCS* Physical Component Summary, *PF* physical functioning, *PsA* psoriatic arthritis, *SD* standard deviation, *SF-36* Short Form-36 Health Survey, *WLQ* Work Limitations Questionnaire

Italics indicate the placebo group throughout the table

^a^SF-36 PCS/MCS included 83 (hands only) and 126 patients (hands or feet)

^b^HAQ-DI, FACIT-F, and arthritis pain (patients with baseline DSS = 0) included 110 patients

^c^Dactylitis was defined as swelling of an entire digit; dactylitis digit counts were evaluated by a blinded, qualified assessor, and severity ranged from 0 to 3 (0 = no tenderness; 3 = extreme tenderness); DSS (sum of scores) ranged from 0 to 60 (60 = highest dactylitis severity) [20]

^d^PASDAS (patients with baseline DSS > 0) included 78–86 patients for hands only, 86–97 patients for feet only, 46–54 patients for hands and feet, and 118–126 patients for hands or feet, across groups

^e^PASDAS (patients with baseline DSS = 0) included 103–113 patients across groups

^f^SF-36 PCS/MCS (patients with baseline DSS = 0) included 108 patients

^g^SF-36 PF (patients with baseline DSS = 0) included 109, 110, and 114 patients receiving tofacitinib 5 mg BID, 10 mg BID, and placebo, respectively

^h^Arthritis pain was measured by 0–100 mm VAS; patients with baseline DSS = 0 included 110 patients receiving tofacitinib 5 mg BID

^i^WLQ dimension sub-scores (patients with baseline DSS > 0) included 44–52 patients for hands only, 51–64 patients for feet only, 29–34 patients for hands and feet, and 68–82 patients for hands or feet, across groups

^j^WLQ dimension sub-scores (patients with baseline DSS = 0) included 63–72 patients across groups

### DSS and dactylitic digits count in patients with DSS > 0 at baseline

**Fig. 1:**
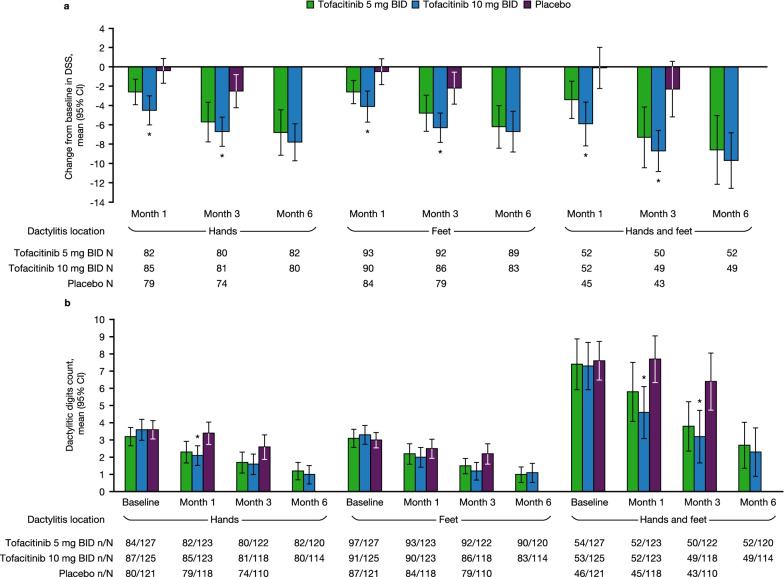
Change from baseline in DSS and dactylitic digits count (patients with baseline DSS > 0). Data for **a** DSS and **b** dactylitis digit count were stratified by dactylitis location and pooled from OPAL Broaden and OPAL Beyond. *Comparisons where the 95% CI for tofacitinib does not overlap with the 95% CI for placebo. Dactylitis was defined as swelling of an entire digit; DSS ranged from 0 to 60 (60 = highest dactylitis severity) [20]. *BID* twice daily, *CI* confidence interval, *DSS* Dactylitis Severity Score, *N* total number of patients with DSS > 0 at baseline, *n* number of patients applicable for each category

Regardless of location (hands only, feet only, hands and feet), improvements from baseline in DSS were greater at months 1 and 3 in patients receiving tofacitinib 10 mg BID, compared with placebo (Fig. 1a). Likewise, in the hands and feet combined group, improvements in dactylitic digit count were greater at months 1 and 3 in patients receiving tofacitinib 10 mg BID, compared with placebo (Fig. 1b). Greater improvements were also observed in the hands only group at month 1 in patients receiving tofacitinib 10 mg BID compared with placebo (Fig. 1b). For both endpoints, improvements with tofacitinib were maintained to month 6.

### Assessment of dactylitis over 6 months in patients with DSS > 0 at baseline, stratified by location

**Fig. 2:**
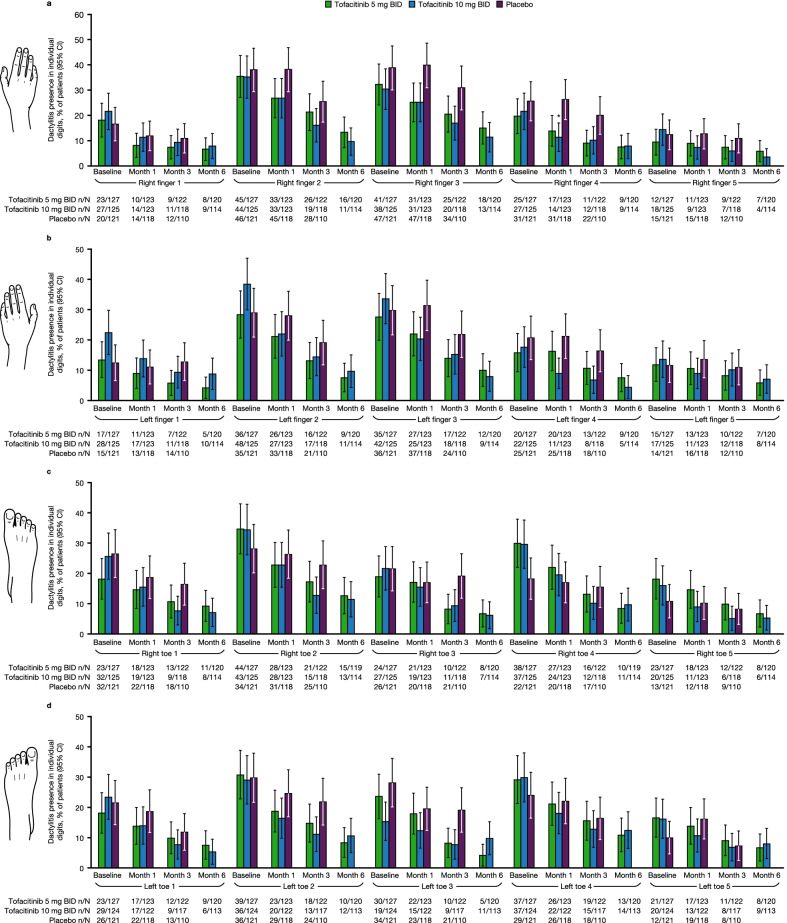
Proportion of patients with dactylitis stratified by location (patients with baseline DSS > 0). Data for **a** right hand fingers, **b** left hand fingers, **c** right foot toes, and **d** left foot toes were pooled from OPAL Broaden and OPAL Beyond. *Comparisons where the 95% CI for tofacitinib does not overlap with the 95% CI for placebo. Dactylitis was defined as swelling of an entire digit; DSS ranged from 0 to 60 (60 = highest dactylitis severity) [20]. *BID* twice daily, *CI* confidence interval, *DSS* Dactylitis Severity Score, *N* total number of patients with DSS > 0 at baseline, *n* number of patients applicable for each category

At baseline, a larger proportion of patients experienced dactylitis in the 2nd and 3rd fingers of the right and left hands, compared with other fingers of either hand (Fig. 2a, b), and the 2nd toes of either foot, compared with other toes (Fig. 2c, d). The proportion of patients with dactylitis decreased following treatment with tofacitinib, and, at month 1, greater improvements were observed in the 4th finger of the right hand with 10 mg BID compared with placebo (Fig. 2a).

At baseline, for all digits in the hands and feet in those receiving tofacitinib 5 and 10 mg BID, dactylitis was present in up to 35.4% and 38.4% of patients, respectively. Improvements in dactylitis presence for individual digits were similar with tofacitinib 5 or 10 mg BID (≤ 15% of patients at month 6) (Fig. 2a–d).

### Development of dactylitis over 6 months in patients with DSS = 0 at baseline, stratified by location

For those with no dactylitis (DSS = 0) at baseline receiving either tofacitinib or placebo, dactylitis had developed in ≥ 1 digits in < 2% of patients at month 1, and up to 3.7% of patients at month 3. Emerging dactylitis was observed in < 2% across treatment groups at month 6 (Additional file 1: Fig. S1a–d).

At month 3, in general, in patients without dactylitis at baseline, few differences were observed in the development of dactylitis in those who received tofacitinib, compared with placebo. Across treatment groups, development of dactylitis through month 6 was more common in the right hand, compared with the left hand, and primarily affected the 1st, 2nd, and 3rd digits (Additional file 1: Fig. S1a, b). Development of dactylitis was more common in the digits of the left foot, compared with the right foot (Additional file 1: Fig. S1c, d).

### Disease activity in patients with DSS > 0 and DSS = 0 at baseline

**Fig. 3:**
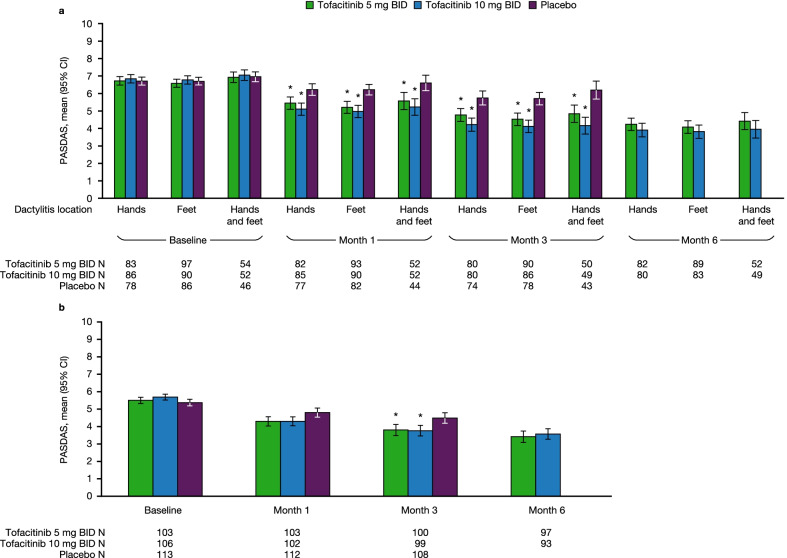
PASDAS by dactylitis location in patients with DSS > 0 or DSS = 0 at baseline. Data for patients with **a** DSS > 0 and **b** DSS = 0 at baseline were pooled from OPAL Broaden and OPAL Beyond. *Comparisons where the 95% CI for tofacitinib does not overlap with the 95% CI for placebo. Dactylitis was defined as swelling of an entire digit; DSS ranged from 0 to 60 (60 = highest dactylitis severity) [20]. *BID* twice daily, *CI* confidence interval, *DSS* Dactylitis Severity Score, *N* total number of patients, *PASDAS* Psoriatic Arthritis Disease Activity Score

In patients with DSS > 0 at baseline, PASDAS was lower with both doses of tofacitinib versus placebo at months 1 and 3, regardless of dactylitis site. PASDAS in patients with dactylitis was 5.2–5.6 and 5.0–5.2 with tofacitinib 5 and 10 mg BID across dactylitis sites, respectively, versus 6.2–6.6 with placebo, at month 1; and 4.5–4.8 and 4.1–4.2 with tofacitinib 5 and 10 mg BID, respectively, versus 5.7–6.2 with placebo, at month 3. Lower PASDAS with tofacitinib was maintained to month 6 (Fig. 3a).

In patients with DSS = 0 at baseline, PASDAS was lower at month 3 with tofacitinib 5 mg BID (mean 3.81 [95% CI 3.49–4.13]) and tofacitinib 10 mg BID (3.76 [95% CI 3.46–4.06]) compared with placebo (4.49 [95% CI 4.19–4.79]). Improvements with tofacitinib were maintained to month 6 (Fig. 3b).

### PROs in patients with DSS > 0 at baseline

**Fig. 4:**
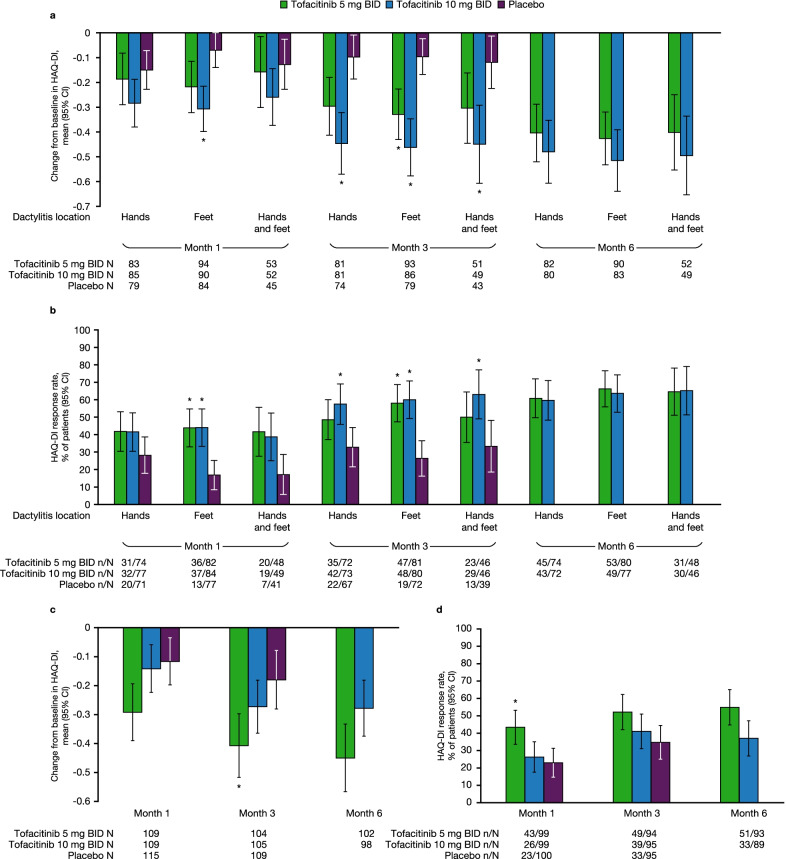
Change from baseline in HAQ-DI scores and response rates. Change from baseline data in **a** HAQ-DI score and **b** HAQ-DI response rate^†^ by dactylitis location in patients with DSS > 0 at baseline; and **c** HAQ-DI score and **d** HAQ-DI response rate^†^, in patients with DSS = 0 at baseline were pooled from OPAL Broaden and OPAL Beyond. *Comparisons where the 95% CI for tofacitinib does not overlap with the 95% CI for placebo. ^†^Defined as ≥ 0.35-point decrease from baseline in HAQ-DI score. Dactylitis was defined as swelling of an entire digit; DSS ranged from 0 to 60 (60 = highest dactylitis severity) [20]. *BID* twice daily, *CI* confidence interval, *DSS* Dactylitis Severity Score, *HAQ-DI* Health Assessment Questionnaire-Disability Index, *N* total number of patients, *n* number of patients applicable for each category

In patients receiving tofacitinib 5 or 10 mg BID, change from baseline in HAQ-DI scores and HAQ-DI response rates are shown in Fig. 4a and b, respectively, and other PRO scores are shown in Additional file 2: Fig. S2.

Regardless of dactylitis site, mean improvements from baseline in HAQ-DI score and HAQ-DI response rate were greater at month 3 with tofacitinib 10 mg BID, compared with placebo (Fig. 4a, b). In the feet only group, greater improvements in HAQ-DI score and HAQ-DI response rate were also observed with both doses of tofacitinib at month 3 (Fig. 4a, b). At month 1, in the feet only group, greater improvements in HAQ-DI score and HAQ-DI response rate were observed with tofacitinib 10 mg BID and both doses of tofacitinib, respectively, compared with placebo (Fig. 4a, b).

Similarly, arthritis pain VAS scores were lower with both doses of tofacitinib versus placebo at month 3, regardless of dactylitis site and were maintained to month 6. At month 1, regardless of location, lower arthritis pain VAS scores were observed with tofacitinib 10 mg BID compared with placebo (Additional file 2: Fig. S2e). In the feet only group at month 1, lower arthritis pain VAS scores were also observed with tofacitinib 5 mg BID compared with placebo (Additional file 2: Fig. S2e).

Mean WLQ time management and output demands scores were lower with tofacitinib 10 mg BID in the hands only and feet only groups, compared with placebo at month 3 (first post-baseline assessment of WLQ dimension scores) (Additional file 3: Fig. S3a, d). Regardless of dactylitis site, mean WLQ physical demands scores were similar across treatment groups at month 3, whereas mean WLQ mental/interpersonal demands scores were lower with tofacitinib 10 mg BID, versus placebo in the hands only group (Additional file 3: Fig. S3b, c). Responses with tofacitinib were generally maintained to month 6, regardless of dactylitis site (Additional file 3: Fig. S3a–d).

Multivariate linear regression analyses demonstrated that dactylitis location was not significantly associated with change from baseline in HAQ-DI, SF-36 PF, or WLQ dimension sub-scores at months 3 and 6 in patients with DSS > 0 at baseline, irrespective of tofacitinib dose; the only parameters that were associated with change from baseline in HAQ-DI, SF-36 PF, or WLQ dimension sub-scores were the baseline scores for each respective PRO. It was observed that in most cases, more unfavorable baseline scores predicted a bigger mean change from baseline in responses.

### PROs in patients with DSS = 0 at baseline

PRO results are shown in Fig. 4c, d, Additional file 4: Fig. S4, and Additional file 5: Fig. S5, including mean WLQ time management, physical demands, mental/interpersonal demands, and output demands scores.

Mean changes from baseline in HAQ-DI score were greater with tofacitinib 5 mg BID, compared with placebo, at month 3 (Fig. 4c), and HAQ-DI response rates were greater with tofacitinib 5 mg BID at month 1 (Fig. 4d).

Mean FACIT-F total scores, SF-36 PCS, SF-36 MCS, and PF scores were similar across treatment groups at months 1 and 3 with both doses of tofacitinib, compared with placebo (Additional file 4: Fig. S4a–d).

Arthritis pain scores in patients with DSS = 0 at baseline were 41.2, 38.1, and 43.7 with tofacitinib 5 mg BID, tofacitinib 10 mg BID, and placebo, respectively, at month 1; and 34.8, 33.0, and 41.4 with tofacitinib 5 mg BID, tofacitinib 10 mg BID, and placebo, respectively, at month 3 (Additional file 4: Fig. S4e).

## Discussion

Phase III RCTs of patients with active PsA who had an inadequate response to csDMARDs (OPAL Broaden), or TNFi (OPAL Beyond), reported the effectiveness of tofacitinib with regard to various articular and patient-reported outcomes in PsA, and reported improvements in several psoriatic disease domains [17, 18]. Greater improvements in DSS were reported with tofacitinib, versus placebo, at month 3 [17, 18].

In this analysis of pooled data from OPAL Broaden and OPAL Beyond, greater improvements in dactylitis were observed among patients receiving tofacitinib 10 mg BID (recommended dosage: 5 mg BID [15, 16]) with pre-existing dactylitis (DSS > 0) at baseline, compared with placebo. Improvements in dactylitis with tofacitinib were observed as early as month 1 (first post-baseline assessment) and maintained up to month 6. PASDAS was lower at month 3 with tofacitinib versus placebo in patients with DSS > 0 (regardless of dactylitis site). We also assessed if location of dactylitis could impact on improvements in PROs; dactylitis location was not significantly associated with PROs (change from baseline in HAQ-DI, SF-36 PF, or WLQ dimension sub-scores). In those patients without pre-existing dactylitis (DSS = 0) at baseline, the proportions of patients developing dactylitis in any digit were low, and were similar with tofacitinib and placebo; however, PASDAS was lower with tofacitinib versus placebo at month 3, and scores were maintained through to month 6.

Dactylitis is more common in the feet, compared with the hands [6, 8], and it is possible that toe dactylitis may be less responsive to therapy than finger dactylitis. Notably, in this analysis when stratified by location, we only observed greater improvements in the 4th finger of the right hand with tofacitinib 10 mg BID at month 1.

Of note, in patients without pre-existing dactylitis at baseline, dactylitis developed in up to 3.7% of patients in the digits of the hands or feet up to month 3, and < 2% developed dactylitis up to month 6, regardless of treatment allocation.

As noted above, in patients with DSS > 0 at baseline, absolute PASDAS score was lower with tofacitinib versus placebo, regardless of dactylitis location. Dactylitic digits are often tender and painful, and this can impact on the physical functioning of patients with PsA. PASDAS is a composite measure of disease activity, which includes assessment of dactylitis [22]. It is possible that changes in dactylitis will correspond with changes in tender/swollen joint counts; thus, treatments that improve dactylitis could also result in improvements in tender/swollen joints, contributing to reductions in PASDAS. Therefore, dactylitis may be acting as an indicator of disease severity in this analysis.

Dactylitis should be considered when treatment options are being evaluated for PsA [6, 7, 10]; however, clinical data on the effects of treatments for PsA on changes in dactylitis have, to date, been limited. An analysis of pooled data from two phase III studies found that treatment with ixekizumab resulted in significant improvements in dactylitis, compared with placebo over 24 weeks [32]. In an analysis of patients with PsA who had an inadequate response to prior therapy, the proportion of patients with active dactylitis was significantly lower after 12 weeks of adalimumab treatment [14]. In addition, a multivariate analysis of predictors of response to treatment in patients with PsA found that 12-month treatment with TNFi, versus DMARDs, was a significant predictor of improvement in dactylitis [7]. An analysis into the effect of csDMARDs or bDMARDs found that only infliximab was effective for the treatment of dactylitis [12, 13]. A review found that a range of treatments (including adalimumab, apremilast, ixekizumab, and ustekinumab) were effective for dactylitis, while abatacept, secukinumab, clazakizumab, and tofacitinib were considered “promising” [33]. Another analysis showed ustekinumab, certolizumab, and infliximab were likely to be efficacious for dactylitis [34]. Therefore, the findings of this current analysis provide additional detail on the effects of tofacitinib treatment on dactylitis in patients with PsA.

This analysis had several limitations. Firstly, this was a post-hoc analysis of pooled data from patients with PsA enrolled in OPAL Broaden and OPAL Beyond; the analysis was limited by the low number of patients in the dactylitis groups, and the effects of tofacitinib were only assessed up to month 6. A further limitation was the method used to assess dactylitis; inter- and intra-observer reliability for clinical assessment of dactylitis can be poor, particularly for the toes [20]. Similarly, inter-observer reliability of the degree of tenderness, which was used in this study to grade the severity of dactylitis, can also be poor [20]. Correspondingly, data describing the baseline distribution of both dactylitis digits count and DSS were skewed to the right, indicating that most patients had lower values for the affected dactylitis digits count and, thus, lower values of DSS, which may have impacted the study results. In addition, no formal statistical testing was carried out to determine differences between treatment groups for dactylitis by location and changes from baseline in PROs. Tofacitinib treatment groups were compared, descriptively, with placebo using 95% CIs.

## Conclusion

In summary, in this post-hoc analysis of pooled data from two phase III studies, treatment with tofacitinib resulted in lasting improvements in dactylitis, with minimal emergence of new dactylitis up to month 6, irrespective of dactylitis location. PASDAS by dactylitis location was lower at month 3 with tofacitinib compared with placebo, and was maintained up to month 6. These results suggest that tofacitinib treatment may benefit those patients with PsA experiencing dactylitis, thus further supporting the use of tofacitinib as a treatment for PsA. Further analyses of data collected over longer time periods are required to further assess the effects of tofacitinib on dactylitis in patients with PsA.

## Supplementary Information

**Additional file 1: Fig. S1.** Proportion of patients without dactylitis (DSS = 0) at baseline who developed dactylitis

**Additional file 2: Fig. S2.** Patient reported outcomes in patients with DSS > 0 at baseline, by dactylitis location

**Additional file 3: Fig. S3.** WLQ scores in patients with DSS > 0 at baseline, by dactylitis location

**Additional file 4: Fig. S4.** Patient-reported outcomes in patients without dactylitis (DSS = 0) at baseline

**Additional file 5: Fig. S5.** WLQ scores in patients without dactylitis (DSS = 0) at baseline

## Data Availability

Upon request, and subject to review, Pfizer will provide the data that support the findings of this study. Subject to certain criteria, conditions, and exceptions, Pfizer may also provide access to the related individual de-identified participant data. See https://www.pfizer.com/science/clinical-trials/trial-data-and-results for more information.

## Abbreviations

bDMARD: Biologic disease-modifying antirheumatic drug
BID: Twice daily
CI: Confidence interval
csDMARD: Conventional synthetic disease-modifying antirheumatic drug
DMARD: Disease-modifying antirheumatic drug
DSS: Dactylitis severity score
FACIT-F: Functional Assessment of Chronic Illness Therapy-Fatigue
HAQ-DI: Health Assessment Questionnaire-Disability Index
HRQoL: Health-related quality of life
IL: Interleukin
MCS: Mental Component Summary
PASDAS: Psoriatic Arthritis Disease Activity Score
PCS: Physical Component Summary
PF: Physical functioning
PRO: Patient-reported outcome
PsA: Psoriatic arthritis
RCT: Randomized controlled trial
SF-36: Short Form-36 Health Survey
TNFi: Tumor necrosis factor inhibitor
VAS: Visual Analog Scale
WLQ: Work Limitations Questionnaire
