# Supplementary material for: Effect of tofacitinib on dactylitis and patient-reported outcomes in patients with active psoriatic arthritis: post-hoc analysis of phase III studies
Source: BMC Rheumatol. 2022 Sep 1;6:68. doi: 10.1186/s41927-022-00298-4 (PMC9434913; doi:10.1186/s41927-022-00298-4)
Supplement: Supplementary file 1 — Additional file 1: Fig. S1. Proportion of patients without dactylitis (DSS = 0) at baseline who developed dactylitis [file 41927_2022_298_MOESM1_ESM.pdf]

Additional file 1: Fig. S1 Proportion of patients without dactylitis (DSS=0) at baseline who developed dactylitis

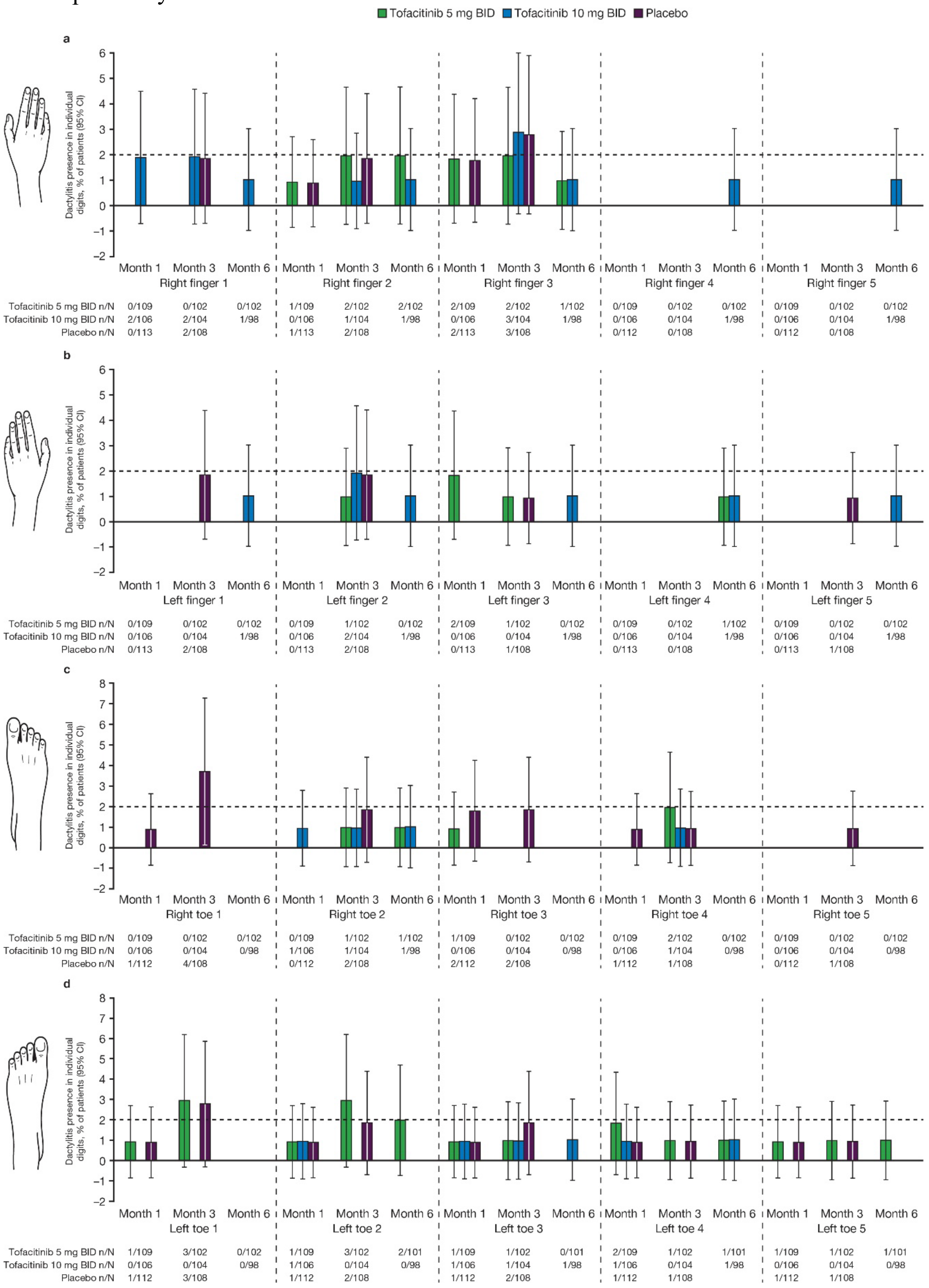

Data stratified by *de novo* dactylitis location for **(a)** right hand fingers, **(b)** left hand fingers, **(c)** right foot toes, and **(d)** left foot toes were pooled from OPAL Broaden and OPAL Beyond.

The dashed line is included to highlight 2% of patients with dactylitis in individual digits.
Dactylitis was defined as swelling of an entire digit; DSS ranged from 0–60 (60=highest dactylitis severity) (Helliwell PS, et al. J Rheumatol 2005;32:1745–50).
BID twice daily, *CI* confidence interval, *DSS* Dactylitis Severity Score, *N* total number of patients with DSS=0 at baseline, *n* number of patients applicable for each category.
