# Supplementary material for: Effect of tofacitinib on dactylitis and patient-reported outcomes in patients with active psoriatic arthritis: post-hoc analysis of phase III studies
Source: BMC Rheumatol. 2022 Sep 1;6:68. doi: 10.1186/s41927-022-00298-4 (PMC9434913; doi:10.1186/s41927-022-00298-4)
Supplement: Supplementary file 2 — Additional file 2: Fig. S2. Patient reported outcomes in patients with DSS > 0 at baseline, by dactylitis location [file 41927_2022_298_MOESM2_ESM.pdf]

**Additional file 2: Fig. S2** Patient reported outcomes in patients with DSS>0 at baseline, by dactylitis location

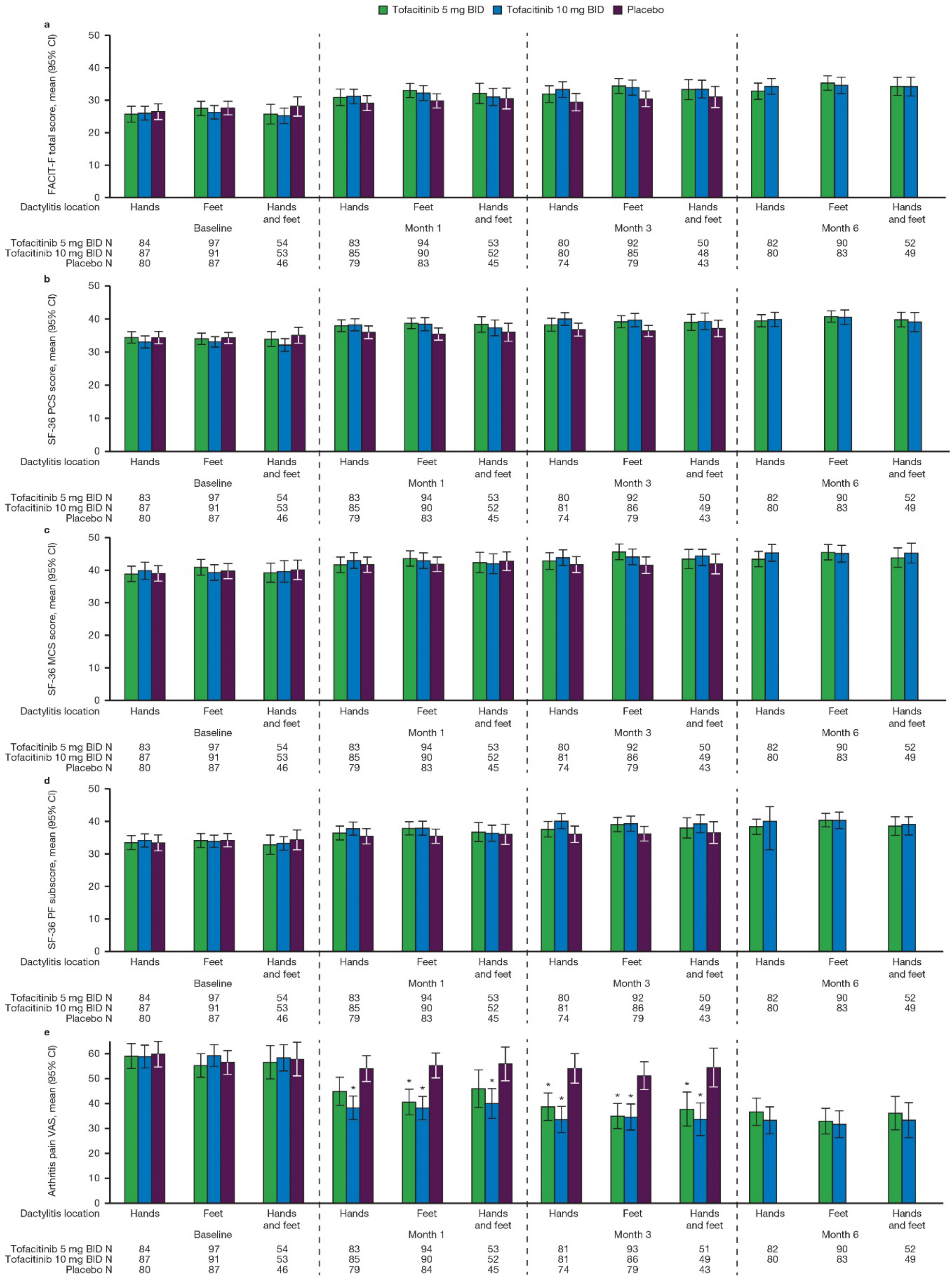


Data for **(a)** FACIT-F total score, **(b)** SF-36 PCS score, **(c)** SF-36 MCS score, **(d)** SF-36 PF sub-score, and **(e)** arthritis pain VAS were pooled from OPAL Broaden and OPAL Beyond.

[*]Comparisons where the 95% CI for tofacitinib does not overlap with the 95% CI for placebo.
Dactylitis was defined as swelling of an entire digit; DSS ranged from 0–60 (60=highest dactylitis severity) (Helliwell PS, et al. J Rheumatol 2005;32:1745–50).
*BID* twice daily, *CI* confidence interval, *DSS* Dactylitis Severity Score, *FACIT-F* Functional Assessment of Chronic Illness Therapy-Fatigue, *MCS* Mental Component Summary, *N* total number of patients with DSS>0 at baseline, *PCS* Physical Component Summary, *PF* physical functioning, *SF-36* Short Form-36 Health Survey, *VAS* Visual Analog Scale.
