# Supplementary material for: Effect of tofacitinib on dactylitis and patient-reported outcomes in patients with active psoriatic arthritis: post-hoc analysis of phase III studies
Source: BMC Rheumatol. 2022 Sep 1;6:68. doi: 10.1186/s41927-022-00298-4 (PMC9434913; doi:10.1186/s41927-022-00298-4)
Supplement: Supplementary file 3 — Additional file 3: Fig. S3. WLQ scores in patients with DSS > 0 at baseline, by dactylitis location [file 41927_2022_298_MOESM3_ESM.pdf]

Additional file 3: Fig. S3 WLQ scores in patients with DSS>0 at baseline, by dactylitis location

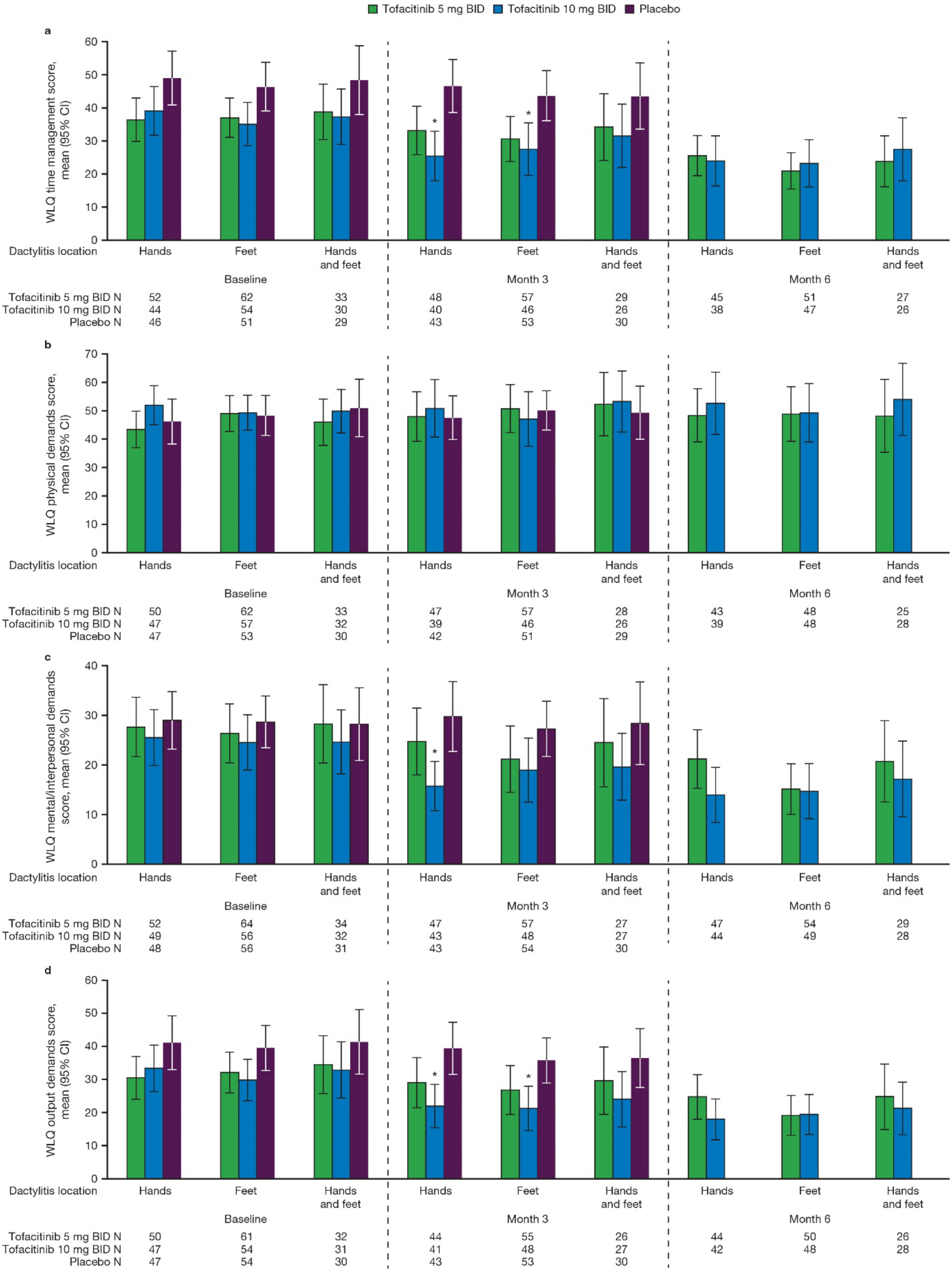

Data for **(a)** WLQ time management score, **(b)** WLQ physical demands score, **(c)** WLQ mental/interpersonal demands score, and **(d)** WLQ output demands score were pooled from OPAL Broaden and OPAL Beyond.

[*]Comparisons where the 95% CI for tofacitinib does not overlap with the 95% CI for placebo.
Dactylitis was defined as swelling of an entire digit; DSS ranged from 0–60 (60=highest dactylitis severity) (Helliwell PS, et al. J Rheumatol 2005;32:1745–50).
BID twice daily, *CI* confidence interval, *DSS* Dactylitis Severity Score, *N* total number of patients with DSS>0 at baseline, *WLQ* Work Limitations Questionnaire.
