# Supplementary material for: Effect of tofacitinib on dactylitis and patient-reported outcomes in patients with active psoriatic arthritis: post-hoc analysis of phase III studies
Source: BMC Rheumatol. 2022 Sep 1;6:68. doi: 10.1186/s41927-022-00298-4 (PMC9434913; doi:10.1186/s41927-022-00298-4)
Supplement: Supplementary file 4 — Additional file 4: Fig. S4. Patient-reported outcomes in patients without dactylitis (DSS = 0) at baseline [file 41927_2022_298_MOESM4_ESM.pdf]

Additional file 4: Fig. S4 Patient-reported outcomes in patients without dactylitis (DSS=0) at baseline

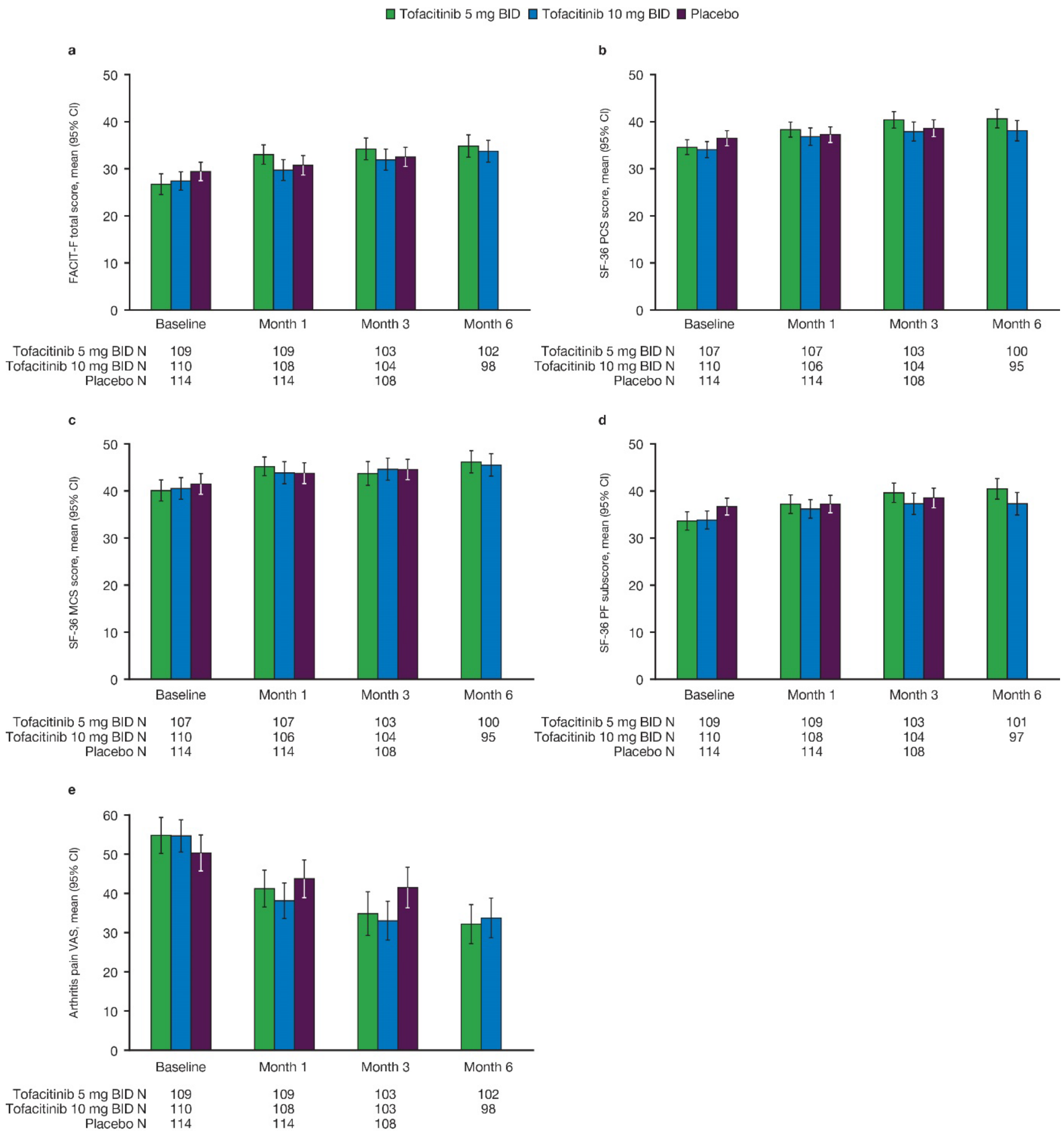

Data for **(a)** FACIT-F total score, **(b)** SF-36 PCS score, **(c)** SF-36 MCS score, **(d)** SF-36 PF sub-score, and **(e)** arthritis pain VAS were pooled from OPAL Broaden and OPAL Beyond.
BID twice daily, *CI* confidence interval, *DSS* Dactylitis Severity Score, *FACIT-F* Functional Assessment of Chronic Illness Therapy-Fatigue, *MCS* Mental Component Summary, *N* total number of patients with DSS=0 at baseline, *PCS* Physical Component Summary, *PF* physical functioning, *SF-36* Short Form-36 Health Survey, *VAS* Visual Analog Scale.
