# Supplementary material for: Effect of tofacitinib on dactylitis and patient-reported outcomes in patients with active psoriatic arthritis: post-hoc analysis of phase III studies
Source: BMC Rheumatol. 2022 Sep 1;6:68. doi: 10.1186/s41927-022-00298-4 (PMC9434913; doi:10.1186/s41927-022-00298-4)
Supplement: Supplementary file 5 — Additional file 5: Fig. S5. WLQ scores in patients without dactylitis (DSS = 0) at baseline [file 41927_2022_298_MOESM5_ESM.pdf]

Additional file 5: Fig. S5 WLQ scores in patients without dactylitis (DSS=0) at baseline

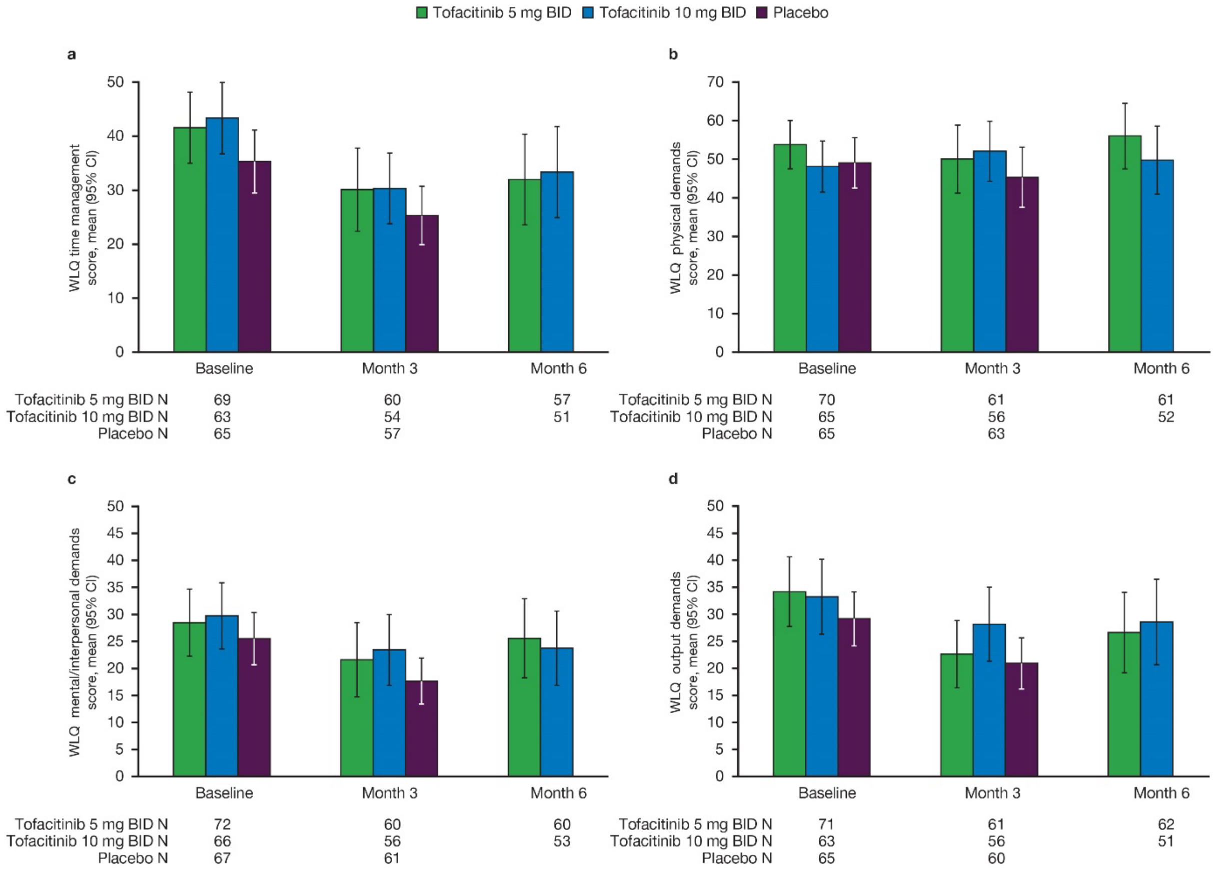

Data for **(a)** WLQ time management score, **(b)** WLQ physical demands score, **(c)** WLQ mental/interpersonal demands score, and **(d)** WLQ output demands score were pooled from OPAL Broaden and OPAL Beyond. *BID* twice daily, *CI* confidence interval, *DSS* Dactylitis Severity Score, *N* total number of patients with DSS=0 at baseline, *WLQ* Work Limitations Questionnaire.
